# Cortical and subcortical auditory evoked potentials with verbal stimulus: correlation and association in adults

**DOI:** 10.1590/2317-1782/e20240301en

**Published:** 2026-01-30

**Authors:** Christine Grellmann Schumacher, Tainá Betti, Hélinton Goulart Moreira, Dayane Domeneghini Didoné, Michele Vargas Garcia

**Affiliations:** 1 Departamento de Fonoaudiologia, Centro de Ciências da Saúde, Universidade Federal de Santa Maria – UFSM - Santa Maria (RS), Brasil.

**Keywords:** Hearing, Electrophysiology, Evoked Potentials, Young Adult, Association, Correlation of Data

## Abstract

**Purpose:**

To analyze the correlation and association between the Long Latency Auditory Evoked Potential (LLAEP) and the Frequency Following Response (FFR) in young adults with normal hearing thresholds.

**Methods:**

This was a cross-sectional, quantitative, and qualitative study. The sample included 32 young adults (mean age of 22.5 years) of both sexes who met the inclusion criteria. The participants underwent basic audiological evaluation, screening of auditory skills through the Random Gap Detection Test and Dichotic Digits Test, and electrophysiological tests: Auditory Brainstem Response with click stimulus, Long Latency Auditory Evoked Potential, and Frequency Following Response with verbal stimulus.

**Results:**

A statistically significant and positive brightness was observed between waves V, A and C and waves P1 and N2, evidencing the participation of auditory structures of the primary auditory cortex in the generation of FFR responses, and a negative appearance between waves C and N2, reflecting the different auditory abilities to generate the responses of each component. There was no significant association between individuals classified as normal and altered in the tests in general performed in the present study or when associated between each component.

**Conclusion:**

Waves V, A, and C correlate with waves P1 and N2 of the Long Latency Auditory Evoked Potential in young adults. There was no evidence of associations between the qualitative results of the Frequency Following Response and the Long Latency Auditory Evoked Potential.

## INTRODUCTION

The ability to identify and comprehend a sound stimulus, whether it is verbal or not, is a task that requires structural integrity and functionality of the central auditory pathway. The Central Auditory Nervous System (CANS) conducts auditory information to the auditory cortex through synaptic activity, and its evaluation is performed objectively through Auditory Evoked Potentials (AEPs). Complex sounds, such as speech sounds, demand greater diligence from the central auditory system, being processed in higher regions such as subcortical and cortical areas^(1)^.

For the assessment at thalamo-cortical structures, primary auditory cortex, associative cortical areas, and frontal cortex^(2,3)^, we have the Long Latency Auditory Evoked Potential (LLAEP). This potential reflects the central processing of sound stimuli as well as attention, memory, and auditory discrimination abilities^(4,5)^. They are divided into exogenous/cortical components (influenced by the characteristics of the stimulus), including P1, N1, P2, mixed N2 component, and endogenous/cognitive component (P300)^(6)^. The P300 reflects cognitive abilities such as attention to the stimulus, discrimination, selection, memory, and decision-making, and is associated with conscious perception of changes in the auditory stimulus^(2)^.

With scientific advancements, new tools emerge to aid in understanding the process of sound encoding by the CANS, and one of these tools is the Frequency Following Response (FFR) generated from verbal stimulation^(7-10)^. The FFR is responsible for evaluating the entire pathway of the stimulus through the CANS and exposes the process of encoding speech sounds^(11)^ and emerges to provide complementary information about complex stimulus processing, similar to the LLAEP, and to reveal specific biological deficits related to sound encoding.

While other potentials record neural responses in the form of electroencephalogram waveforms, providing only temporal information, the FFR stands out because its waveform reflects its complexity by simulating the acoustic properties of the stimulus and preserving the stimulus formants in the response. This potential captures the smallest changes in the sound signal, such as those observed in consonants, and primarily assesses the processing of the temporal and spectral domains of the stimulus^(12)^.

Although the recording of LLAEP and FFR responses is distinct, the literature^(12-15)^ highlights that part of the FFR responses would originate in the central auditory structures, that is, structures that also generate LLAEP responses. Therefore, the FFR is capable of providing information related to early cortical sound coding activities, justifying the importance of correlation studies between the mentioned potentials.

Based on the potential relationships between the aforementioned potentials, considering the evaluated auditory abilities and regions of neural synapse activation, this research was based on the hypothesis that these potentials have a relationship and do not exhibit qualitative association. Furthermore, it is justified by the need for a better understanding of the FFR for its inclusion in audiological clinical practice. Therefore, this study was designed with the objective of analyzing the correlation and association between the FFR and the LLAEP in young adults with normal hearing thresholds, taking into consideration the latency values of the potential.

The objective of the research was to analyze the correlation and association between the Long Latency Auditory Evoked Potential (LLAEP) and the Frequency Following Response (FFR) in young adults with normal hearing thresholds.

## METHODS

Procedures were carried out in the Speech Pathology and Auditory Electrophysiology Outpatient Service of a teaching hospital. The study was approved by the Research Ethics Committee, CAAE: 23081.019037/2017-19. It had a cross-sectional, quantitative, and qualitative design. The research complied with all norms and guidelines for research involving human subjects outlined in Resolution # 510/16 of the National Health Council^(16)^. All individuals who agreed to participate signed an informed consent form.

The following inclusion criteria were adopted: Individuals aged between 18 and 35 years, who are healthy, native speakers of Brazilian Portuguese, with normal hearing thresholds in both ears (hearing thresholds up to 20 dBHL for frequencies from 250Hz to 8000Hz in both ears)^(17)^, no auditory complaints, type A tympanometry bilaterally (compliance from 0.3 to 1.65ml and pressure up to −100daPa), contralateral acoustic reflexes present at normal levels in both ears (reflections present at frequencies of 500Hz, 1000Hz, 2000Hz and 4000Hz, elicited from 70 to 100dB)^(18)^, normality in screening auditory abilities tested through the Dichotic Digits Test (only for binaural integration)^(19)^ and the Random Gap Detection Test^(20)^ and and normality in the Electrophysiological tests, such as Auditory Brainstem Response (ABR), with normal standards in both ears, with the presence of waves I, III, and V and normal interpeak intervals I-III, III-V, and I-V.

This caseload comprised 32 participants who met the inclusion criteria and volunteered to participate in the study. Among them, 20 (62%) were female and 12 (38%) were male. The mean age was 22.5 years, ranging from 18 to 32 years, with an average education of 14.9.

### Sampling procedures

Subjects were submitted to hearing history-taking, meatoscopy, pure-tone threshold audiometry^(17)^ and acoustic immittance testing^(18)^.

The tests of auditory processing (DDT and RGDT) were also performed on the AD226d audiometer. These tests were applied with the aim of tracking central auditory processing.

The Dichotic Digits Test (only the binaural integration) with four numbers, two in each ear, were simultaneously presented, and the individual was instructed to repeat the numbers heard, regardless of order. The normality criterion used was 95% accuracy in both ears for the age range of the study^(19)^, and Random Gap Detection Test where the individual needed to identify the presence of a gap in pure tones at frequencies of 0.5, 1, 2, and 4 kHz, with random intervals ranging from 0 to 40 ms between the tones. The normality criterion used was the average of frequencies ≤ 10 ms, marked from the moment the subject identified the gap^(20,21)^.

The Auditory Brainstem Response using click stimulus and the FFR test were carried out using Smart EP equipment from Intelligent Hearing Systems (IHS). Tests were performed in a single day and lasted approximately 1 h 30 min.

Prior to the tests, the skin was cleaned with abrasive paste at the electrode attachment sites. The stimuli were presented using insert earphones (ER-3A), with impedance values kept equal to or below 3 KOhms, and the number of artifacts did not exceed 10% of the number of stimuli.

**Figure 1:**
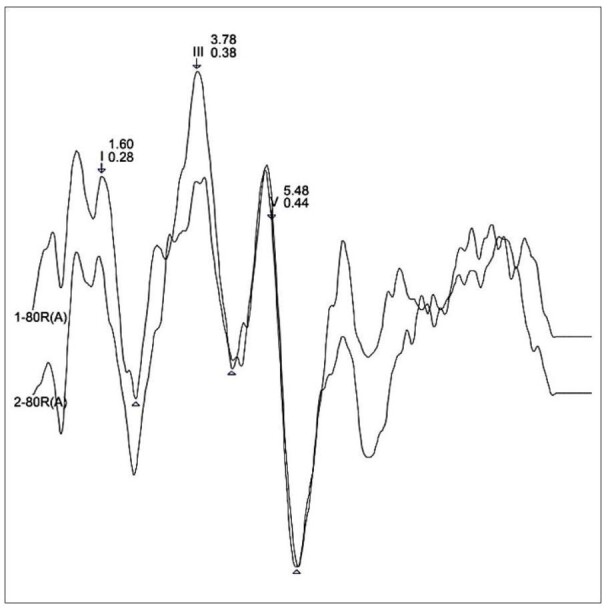
Auditory Brainstem Response (ABR) with click stimulus - waveform recorded in one of the research subjects

The Fz electrode was placed on the central and superior portion of the forehead, the ground electrode (Fpz) on the central and inferior portion of the forehead, and the reference electrodes M1 and M2 on the left and right mastoids. The parameters used for the Brainstem Auditory Evoked Potential were: intensity of 80 dB nHL, with monaural stimulation, first in the right ear, then in the left, with a recording window of 12 ms and a presentation rate of 27.7/s. The stimuli were filtered with a low-pass filter of 3000 Hz and a high-pass filter of 100 Hz, with a repetition rate gain of 100.0 K and a duration of 100 µsec. The polarity used was rarefaction, and two stimulations of 2048 sweeps each were performed. The reference values are as follows: I=1.66 (SD: 0.10); III=3.87 (SD: 0.15); V=5.68 (SD: 0.12); I-III=2.21 (SD: 0.14); III-V=1.8 (SD: 0.10); I-V=4.02 (SD: 13)^(22)^. For the analysis of the waveforms, the morphology, latency, and replicability of waves I, III, and V (Figure 1), as well as the interpeak intervals I-III, III-V, and I-V, were taken into consideration. Subjects were considered to have abnormalities if their latencies were outside the normal range by two standard deviations or if a wave was absent.

The individuals who exhibited normal responses to the aforementioned procedures underwent the following research procedures: Frequency Following Response and Long Latency Auditory Evoked Potential.

**Figure 2:**
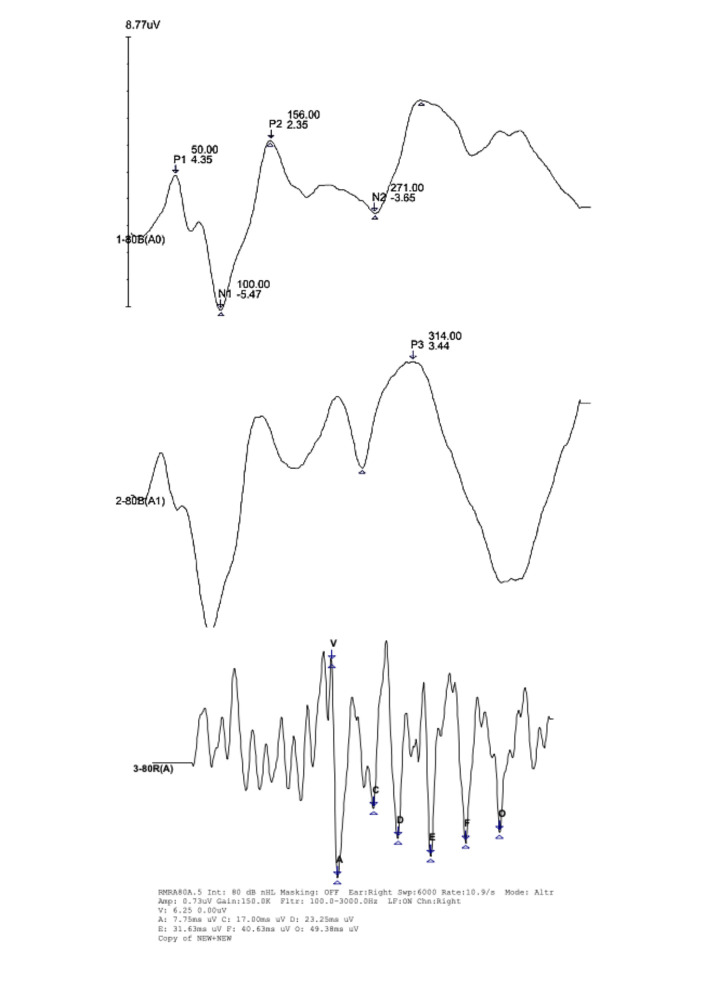
Illustration of the exogenous, mixed, and endogenous components of the Long Latency Auditory Evoked Potential and Frequency-following response - tracing recorded in one of research subjects

**Figure 3:**
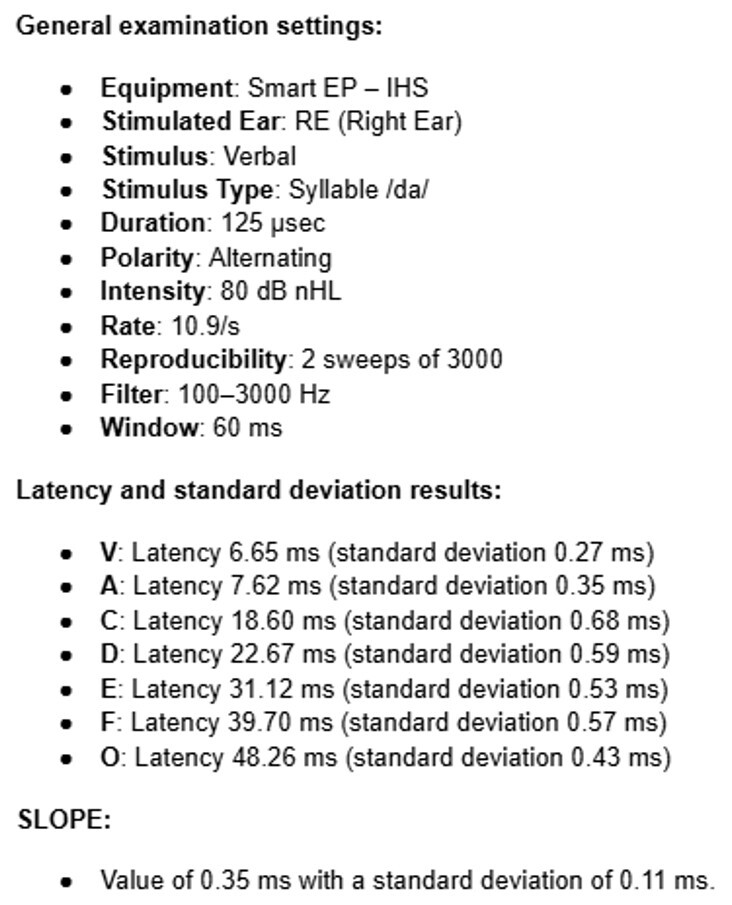
Protocol parameters for *Frequency Following Response*

The Frequency Following Response (Figure 2) was conducted using the same electrode configuration as the Auditory Brainstem Response (ABR), with presentation of stimuli in the right ear (monaural). The parameters are described in Figure 3. The reference values for the test were based on Song et al.^(23)^.

Caption: IHS = Intelligent Hearing Systems; RE = Right Ear; µsec = microseconds; dB nHL = hearing level; s = seconds; Hz = Hertz; ms = milliseconds

Caption: IHS = Intelligent Hearing Systems; RE = Right Ear; LE = Left Ear; dB nHL = hearing level; s = seconds; Hz = Hertz; ms = milliseconds; added with 2SD (standard deviations)

The Slope, in turn, was calculated using the formula Amp V - Amp A / Lat A - Lat V, which was provided by researcher Nina Kraus in direct communication with the authors. Frequency domain analysis was not performed in this study as the MATLAB software required for this analysis is not available on the IHS equipment.

In the Long Latency Auditory Evoked Potential, the Cz electrode was positioned on the cranial vertex, while the other electrodes remained in the same location as the other potentials: Fpz as the ground electrode (on the forehead), and M1 as the left mastoid and M2 as the right mastoid (reference electrodes). The participant was instructed to mentally count the rare stimuli /di/ in a series of frequent stimuli /ba/ presented in an oddball paradigm. Auditory stimuli were presented binaurally. It is important to note that the choice of verbal stimuli for the LLAEP was made according to the availability of the equipment and, mainly, due to the fact that the chosen reference^(24)^ uses the same stimuli and the same equipment.

**Figure 4:**
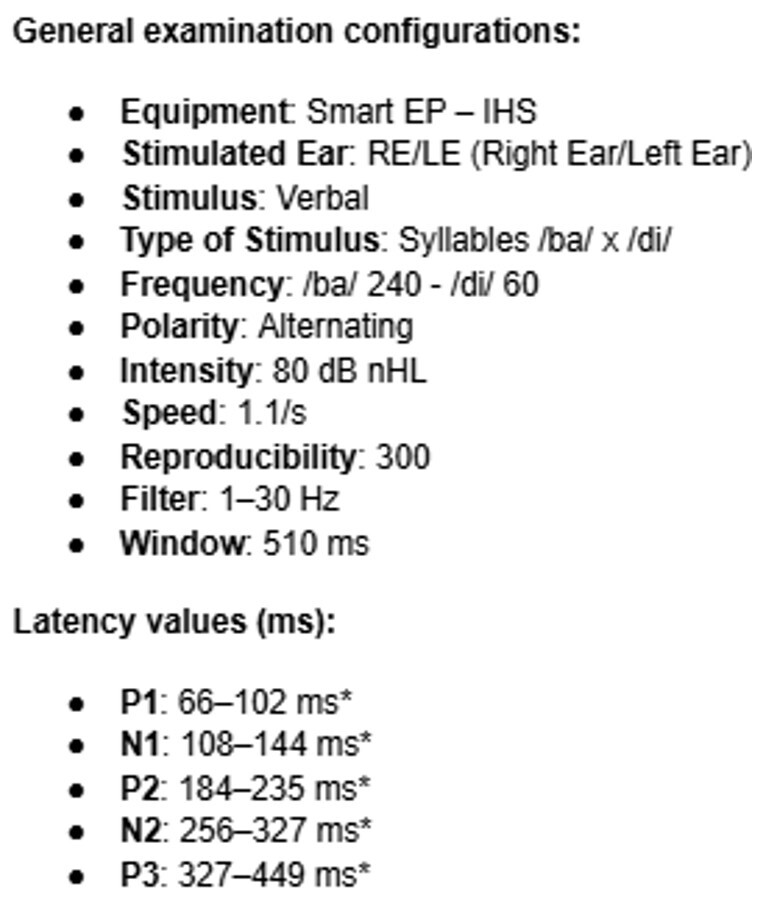
Protocol parameters for Long Latency Auditory Evoked Potential

Analysis of the P1, N1, P2, and N2 waves was performed on the waveform corresponding to the frequent stimuli, while the P3 wave was analyzed in the waveform corresponding to the rare stimuli (Figure 2). The test parameters and reference values for the normality criteria were based on Didoné et al.^(24)^ and are presented in Figure 4. The absence of components was considered as an altered result.

For the statistical analysis, the data were entered into Microsoft Office Excel spreadsheets. The Shapiro-Wilk test was used to assess the normality of the sample. Pearson's correlation test was then employed to analyze the correlation between the components, and Fisher's exact test was used to assess their association. These analyses were performed using the Statistica 7 software. The significance level was set at 5%.

It is worth noting that the correlation and association analyses were performed for each component of the FFR and LLAEP considering the number of patients for whom the waves were identified in the electrophysiological tracings, with the sample number detailed in the tables of this study.

## RESULTS

Table 1 displays the correlation between the components of FFR and LLAEP. The Pearson correlation test revealed a positive correlation between the VA complex and P1 wave, a negative correlation between the VA complex and N2 wave, and a positive correlation between C and N2, all of which were statistically significant.

**Table 1:** Correlation table between FFR × LLAEP components

	N P1		N N1		N P2		N N2		N P3	
V	24	r=0.54	30	r=0.21	30	r=0.04	26	r=-0.47	22	r=-0.33
		*P* = 0.007		*P* = 0.256		*P* = 0.816		*P* = 0.015		*P* = 0.137
A	24	r=0.64	30	r=0.05	30	r=-0.07	26	r=-0.44	22	r=-0.29
		*P* = 0.001		*P* = 0.784		*P* = 0.708		*P* = 0.023		*P* = 0.193
C	22	r=-0.26	28	r=0.06	28	r=0.29	24	r=0.43	20	r=-0.27
		*P* = 0.238		*P* = 0.746		*P* = 0.134		*P* = 0.036		*P* = 0.253
D	18	r=0.11	22	r=0.01	22	r=0.20	19	r=0.08	18	r=-0.08
		*P* = 0.651		*P* = 0.969		*P* = 0.379		*P* = 0.732		*P* = 0.727
E	22	r=0.38	27	r=-0.09	27	r=-0.14	24	r=-0.11	20	r=-0.32
		*P* = 0.078		*P* = 0.622		*P* = 0.497		*P* = 0.605		*P* = 0. 173
F	23	r=0.10	29	r=0.02	29	r=-0.29	25	r=0.12	21	r=0.01
		*P* = 0.634		*P* = 0.918		*P* = 0.119		*P* = 0.557		*P* = 0.973
O	23	r=0.35	28	r=0.11	28	r=0.14	24	r=-0.20	21	r=-0.28
		*P* = 0.105		*P* = 0.596		*P* = 0.489		*P* = 0.356		*P* = 0.213

Caption: N = number of subjects with presence of potential; p = p-value; r = correlation strength. Statistic: Pearson Correlation Test. Bold = statistically significant correlation

Table 2 presents the association between the components of FFR and LLAEP. Fisher's exact test revealed no significant association between the components. Similarly, in Table 3, Fisher's exact test did not show any statistically significant qualitative association between FFR and LLAEP components.

**Table 2:** Association table between normal and altered quantitative components in FFR and LLAEP

FFR		P1			N1			P2			N2			P3		
		N	A	p-value	N	A	p-value	N	A	p-value	N	A	p-value	N	A	p-value
V	N	23	8	0.557	28	3	0.744	27	4	0.701	25	6	0.625	21	10	0.493
		71.88%	25.00%		87.50%	9.38%		84.38%	12.50%		78.13%	18.75%		65.63%	31.25%	
	A	1	0		1	0		1	0		1	0		1	0	
		3.13%	0.00%		3.13%	0.00%		3.13%	0.00%		3.13%	0.00%		3.13%	0.00%	
A	N	19	7	0.601	23	3	0.382	23	3	0.732	22	4	0.310	17	9	0.392
		59.38%	21.88%		71.88%	9.38%		71.88%	9.38%		68.75%	12.50%		53.13	28.13	
	A	5	1		6	0		5	1		4	2		5	1	
		15.63%	3.13%		18.75%	0.00%		15.63%	3.13%		12.50%	6.25%		15.63%	3.13%	
C	N	22	8	0.400	28	2	0.042	27	3	0.098	24	6	0.483	20	10	0.325
		68.75%	25.00%		87.50%	6.25%		84.38%	9.38%		75.00%	18.75%		62.50%	31.25%	
	A	2	0		1	1		1	1		2	0		2	0	
		6.25%	0.00%		3.13%	3.13%		3.13%	3.13%		6.25%	0.00%		6.25%	0.00%	
D	N	14	4	0.681	17	1	0.401	15	3	0.419	16	2	0.209	12	6	0.773
		43.75%	12.50%		53.13%	3.13%		46.88%	9.38%		50.00%	6.25%		37.50%	18.75%	
	A	10	4		12	2		13	1		10	4		10	4	
		31.25%	12.50%		37.50%	6.25%		40.63%	3.13%		31.25%	12.50%		31.25%	12.50%	
E	N	20	4	0.059	22	2	0.726	22	2	0.217	21	3	0.117	16	8	0.660
		62.50%	12.50%		68.75%	6.25%		68.75%	6.25%		65.63%	9.38%		50.00%	25.00%	
	A	4	4		7	1		6	2		5	3		6	2	
		12.50%	12.50%		21.88%	3.13%		18.75%	6.25%		15.63%	9.38%		18.75%	6.25%	
F	N	17	5	0.660	20	2	0.935	20	2	0.387	19	3	0.272	13	9	0.080
		53.13%	15.63%		62.50%	6.25%		62.50%	6.25%		59.38%	9.38%		40.63%	28.13%	
	A	7	3		9	1		8	2		7	3		9	1	
		21.88%	9.38%		28.13%	3.13%		25.00%	3.25%		21.88%	9.38%		28.13%	3.13%	
O	N	18	6	1.000	22	2	0.726	22	2	0.217	20	4	0.601	17	7	0.660
		56.25%	18.75%		68.75%	6.25%		68.75%	6.25%		62.50%	12.50%		53.13%	21.88%	
	A	6	2		7	1		6	2		6	2		5	3	
		18.75%	6.25%		21.88%	3.13%		18.75%	6.25%		18.75%	6.25%		15.63%	9.38%	

Caption: N = normal; A = altered; Statistics: p-value for Fisher's Exact Test

**Table 3:** Association table between normal and altered qualitative components in FFR and LLAEP

		**LLAEP**		**p-value**
		Normal	Altered	0.114
**FFR**	Normal	7	5
		21.88%	15.63%
	Altered	6	14
		18.75%	43.75%

Caption: FFR = Frequency Following Response; LLAEP = Long Latency Auditory Evoked Potential; Statistics: p-value for Fisher's Exact Test

## DISCUSSION

This study stands out for providing further evidence on the relationship between FFR and LLAEP, emphasizing the importance of using complex stimuli in objective tests, as they reveal detailed information about the performance of the Central Auditory Nervous System (CANS) during acoustic signal processing.

Recently, a published study demonstrated the sensitivity and specificity of FFR in evaluating the Central Auditory Processing (CAP) when compared to the Middle Latency Auditory Evoked Potential^(8)^. The participants in this research showed normal Brainstem Auditory Evoked Response (BERA) results, which reinforces and corroborates findings from previous studies^(13,25,26)^, stating that the subcortical and cortical processing of verbal sounds is not directly related to structural issues.

Authors^(13,15)^ had previously argued for the cortical contribution in obtaining FFR responses. The main finding of this study, as shown in Table 1, is the correlation between FFR and LLAEP waves. The positive correlations between the VA complex and the P1 wave suggest that the consonant identification process requires a contribution from the primary auditory cortex, as reflected by the P1 wave, which indicates the arrival of the stimulus at the cortex. When examining the association between these two components (Table 2), it was observed that out of 24 individuals with present responses for both ears, 23 showed normality in V and P1, and 19 for A and P1, with correlation strengths of r=0.54 and r=0.64, respectively. This demonstrates that an increase in VA complex latency may result in an increase in P1 wave latency (as indicated by the correlation strengths), indicating a possible dependency between these components in processing complex stimuli.

It is important to highlight that this association is in line with the functionality of the central auditory structures assessed by the VA components of the FFR and the P1 component of the LLAEP. The VA components of the FFR reflect the onset portion of the syllable /da/, that is, the beginning of coding at the central level^(11)^. The P1 component also reflects the onset of neural coding^(6)^, which may justify the association with correlation strengths of the results observed in the present study.

In the positive correlation (Table 1) between component C, responsible for the transition from consonant to vowel^(27)^, and N2, associated with the interpretation of detection and identification abilities of the stimulus^(28)^, 24 individuals showed normality in both FFR and LLAEP, with a correlation strength of r=0.43. Although there is no qualitative association (Table 2) between the components, the analysis of absolute latencies strongly suggests that an increase in latency of component C may lead to increased latency values of component N2.

The N2 component is considered a mixed potential, intrinsically related to attentional issues^(6)^. Because the C wave of the FFR is related to the neural representation of the detection of the change between the perception of the consonant and vowel^(11,13,15)^, it is believed that the correlation between the two components can probably be related to similar generating sites that are associated with the perception of changes in the patterns of the acoustic stimulus. This fact could justify the findings of the correlation of the N2 component and the C wave of the FFR in the present study.

Furthermore, a negative correlation was observed between the VA complex and the N2 component, with correlation strengths of r=-0.47 and r=-0.44, respectively. This finding reinforces the different abilities during the encoding process, as the VA complex reflects the perception of the consonant without indicating information that may be related to the abilities reflected by the N2 component.

As previously described, the N2 component is related to issues of attention to changes in acoustic stimuli^(6)^, that is, the perception of different stimuli during the LLAEP assessment causes the individual to pay attention to different stimuli during the series of frequent stimuli. Since the VA complex of FFR is related to the perception of the stimulus consonant /da/ (universal syllable used to obtain FFR responses) and there are no changes in this pattern during the examination^(11,13,15)^, there is no need for the individual to pay attention to the stimulus and identify different stimuli, which may justify the negative correlation between the VA complex and the N2 component, despite N2 having been obtained with the oddball paradigm /ba/ and /di/.

Table 3 shows the association between the potentials. For present alterations, an increase in latency (considering two standard deviations) or absence of the potential was considered. Alterations were observed in both tests in 43.75% (14 individuals) of the sample, with no statistically significant association, and without any proven neurobiological alteration or symptoms. This finding was unexpected, considering that procedures were performed to ensure the normality of the subjects; however, alterations were found in some cases.

One possible justification is the use of verbal stimulation for capturing the potential. This finding aligns with the statement made by Silva et al.^(6)^, which suggests that when using speech stimuli, the recognition process becomes more complex, and the speed and quality of auditory processing may be affected. In the mentioned study, delayed findings in the LLAEP are justified due to the complexity of discriminating speech stimuli. It is worth noting that we chose to present the rare stimulus /di/, due to the ease of perceiving the acoustic differences between frequent /ba/ and rare stimuli /di/.

To mitigate possible alterations in evoked potentials, two preventive interventions could have been implemented, considering that the study was conducted with normal individuals. One of these measures would be the use of the Central Auditory Processing Skill Self-Perception Scale (CAPSSPS), a self-assessment questionnaire recently published in 2022^(29)^. This self-perception scale allows participants to report any suspected alterations in temporal resolution and/or auditory closure. Another additional measure to avoid altered findings in the potentials would be the administration of a comprehensive central auditory processing (CAP) evaluation battery. The comprehensive application of the CAP evaluation battery would provide a more complete and detailed assessment of the participants' central auditory processing abilities, allowing for a more precise analysis of the results obtained.

It is worth noting that the analysis performed in this Table 3 is global, meaning that an alteration in just one component indicates an overall alteration. It can be inferred that an alteration in just one component may go unnoticed by the individual, meaning it can be asymptomatic. Furthermore, it may be masked during the assessment of auditory abilities behaviorally, as the Central Auditory Nervous System (CANS) can compensate for some deficits in sound processing through auditory plasticity. Moreover, the capacity of the human brain to change and reorganize with auditory experience is more effective in young individuals^(30)^, which is the population of the present research. It is also worth mentioning that the LLAEP was the last test conducted during data collection in the individuals, and fatigue can interfere with LLAEP responses, as N2 and P3 are influenced by cognitive functions. Another justification may be related to possible changes in auditory skills that were not directly assessed in this study, since it was decided to perform a screening of these skills.

The integration and interactivity of CANS structures involved in acoustic signal processing are advocated by *Kraus and White-Schwoch*^(12)^. Corroborating this information, the present study demonstrates how complex the information generated by the FFR, in its transient and sustained portions, is, confirming its involvement throughout the entire central auditory pathway. In the present study, it was possible to infer the relationship between cortical potentials and FFR (Table 1), as activities such as stimulus detection and spectral and temporal aspects maintain behavior along the central auditory pathway. Studies^(31,32)^ that report changes in FFR after auditory or cognitive skills training are also valid to justify the possible influence of central auditory structures in the generation of these components. Auditory skills training is intrinsically related to changes in central auditory plasticity in cortical regions, due to the strengthening of synapses and the formation of new neural connections. Thus, the changes observed in the FFR in individuals undergoing auditory training may infer the participation of cortical generating sites, that is, cortical changes influence the FFR responses.

Although these findings do not provide information about the specific generator sites of the FFR, they contribute to a better understanding of this potential. Some limitations of this study include the absence of imaging exams to reinforce these findings, as well as the lack of frequency domain analysis using MATLAB. Another limitation is that the study only included a screening of auditory abilities through two tests that address two important skills for speech perception and central processing. Furthermore, a comprehensive battery of central processing assessment, the absence of a self-assessment questionnaire due to the lack of CAPSSPS^(29)^ at the time, and the absence of cognitive screening were also limitations. It is suggested that these factors be included, if possible, in future studies.

## CONCLUSION

The V, A, and C waves show correlation with the P1 and N2 waves of the LLAEP. However, no associations were found between the FFR and the LLAEP, demonstrating the impartiality of each test.
